# Effect of maternal docosahexaenoic acid supplementation during lactation on breast milk DHA concentration and infant neurodevelopment among urban and rural populations of Punjab, India

**DOI:** 10.3389/fnut.2026.1885518

**Published:** 2026-07-14

**Authors:** Richika Gupta, Kiran Bains, Renuka Aggarwal

**Affiliations:** Department of Food and Nutrition, College of Community Science, Punjab Agricultural University, Ludhiana, India

**Keywords:** breast milk, DASII, docosahexaenoic acid, infant growth, lactation, neurodevelopment, Omega-3 fatty acids

## Abstract

Docosahexaenoic acid (DHA) plays a critical role in infant growth and neurodevelopment during early life; however, dietary intake of omega-3 fatty acids among Indian lactating women is often inadequate, potentially compromising maternal DHA status and infant development. The present study evaluated the effects of maternal DHA supplementation during lactation on maternal DHA status, breast milk DHA concentration, infant growth, and neurodevelopment among urban and rural populations in Punjab, India. A community-based randomized supplementation trial was conducted among 60 healthy lactating mothers (1 month postpartum) and their exclusively breastfed infants. Participants were stratified by residence and randomly assigned to urban control (UC), urban supplemented (US), rural control (RC), or rural supplemented (RS) groups (*n* = 15 per group). Supplemented mothers received DHA capsules daily for 150 days. Maternal dietary intake, erythrocyte DHA concentration, breast milk DHA concentration, infant anthropometric measurements, WHO growth indicators, and neurodevelopmental outcomes assessed using the Developmental Assessment Scale for Indian Infants (DASII) were evaluated. Dietary intake of alpha-linolenic acid and DHA was inadequate in both populations and significantly lower among rural mothers (*p* < 0.001). Maternal DHA supplementation significantly increased erythrocyte DHA concentration and breast milk DHA concentration in both supplemented groups (*p* < 0.001). Infants of supplemented mothers demonstrated significantly higher weight, length, and head circumference measurements at 6 months postpartum compared with controls (*p* < 0.05). Supplemented infants also exhibited more favorable weight-for-age, length-for-age, and weight-for-length Z-scores, together with a lower prevalence of slow weight gain. Furthermore, supplemented infants achieved significantly higher Motor Development Quotient and Mental Development Quotient scores than controls (*p* < 0.001). Breast milk DHA concentration was positively associated with infant neurodevelopmental outcomes. These findings suggest that maternal DHA supplementation during lactation was associated with improved maternal DHA status, enhanced breast milk DHA concentration, favorable infant growth indicators, and higher neurodevelopmental scores. Maternal DHA supplementation may therefore represent a useful nutritional strategy for improving maternal and infant health outcomes in populations with low habitual omega-3 fatty acid intake.

## 1 Introduction

Docosahexaenoic acid (DHA), an omega-3 long-chain polyunsaturated fatty acid (n-3 LCPUFA), is essential for brain and retinal development during early life. Rapid DHA accretion occurs during late gestation and the first 2 years after birth, a critical window for neuronal growth, synaptogenesis, myelination, and visual maturation (1, 2). Since DHA is a major structural component of neuronal membranes and retinal photoreceptors, adequate supply during infancy is crucial for optimal cognitive, motor, and behavioral development.

Human milk is the primary source of DHA for exclusively breastfed infants. However, breast milk DHA concentration largely depends on maternal dietary intake and maternal fatty acid status (3). Endogenous conversion of alpha-linolenic acid to DHA is limited in humans; therefore, direct dietary intake from fish, seafood, fortified foods, or supplements becomes important during lactation (4). Previous studies have shown that maternal DHA supplementation can increase breast milk DHA concentration and may improve infant neurodevelopmental outcomes, including visual acuity, attention, and psychomotor performance (5, 6).

Despite its importance, inadequate omega-3 fatty acid intake remains common in many low- and middle-income countries, including India. Indian diets are predominantly cereal-based and often characterized by low fish consumption and high intake of omega-6-rich vegetable oils, which may reduce endogenous DHA synthesis (7). Recent evidence indicates that lactating women in inland and rural Indian populations frequently exhibit low DHA status and suboptimal breast milk DHA concentrations (8, 9). In Punjab, traditional postpartum food practices, limited dietary diversity, and poor awareness regarding omega-3-rich foods may further contribute to inadequate DHA intake among lactating mothers.

Maternal DHA status may influence infant neurodevelopment through modulation of membrane fluidity, neurotransmission, synaptic signaling, and anti-inflammatory pathways (10). However, evidence regarding the impact of maternal DHA supplementation on infant neurodevelopment in Indian populations remains limited, particularly in relation to urban–rural differences in dietary patterns and nutritional status. Furthermore, region-specific data from Punjab are scarce.

Therefore, the present study aimed to evaluate the effect of maternal DHA supplementation during lactation on maternal DHA status, breast milk DHA concentration, and infant neurodevelopmental outcomes among urban and rural populations of Ludhiana district, Punjab.

## 2 Methodology

### 2.1 Study design and participants

This community-based randomized controlled supplementation trial was conducted among urban and rural lactating mothers in Ludhiana district, Punjab, India, between 1 and 6 months postpartum. Five urban wards and five rural villages were randomly selected for participant recruitment through Anganwadi Centres and Civil Hospital, Ludhiana.

**Figure 1:**
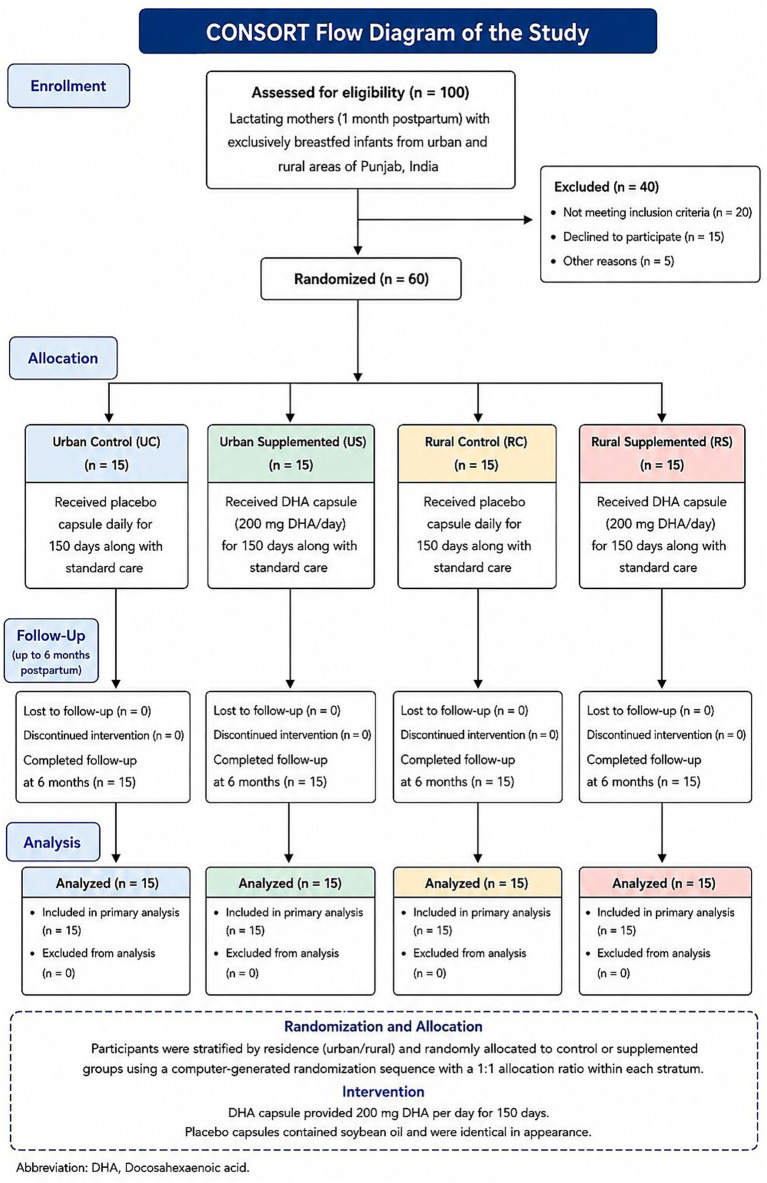
CONSORT flow diagram showing participant screening, eligibility assessment, randomization, allocation, follow-up, and analysis of lactating mothers enrolled in the DHA supplementation trial among urban and rural populations of Ludhiana district, Punjab, India.

Initially, 100 lactating mothers (50 urban and 50 rural) were screened for eligibility. Mothers aged 18–35 years with healthy singleton infants and willingness to participate throughout the study period were considered eligible. Participants with chronic illnesses, metabolic disorders, severe anemia, high-risk pregnancies, previous omega-3 supplementation, or any medical condition likely to affect maternal nutritional status, lactation, infant growth, or neurodevelopment were excluded. Following screening, 60 eligible mothers were enrolled in the study (Figure 1).

The sample size was determined based on feasibility considerations, participant availability, study duration, resource constraints, and the intensive biochemical, anthropometric, and neurodevelopmental assessments required during the intervention period. As the study formed part of a doctoral research programme, a total of 60 participants were recruited and followed longitudinally from 1 to 6 months postpartum. The study was designed as an exploratory intervention trial to generate preliminary evidence regarding the effects of maternal DHA supplementation on breast milk DHA concentration and infant developmental outcomes among urban and rural populations in Punjab.

Following baseline assessment, eligible urban and rural participants were randomly allocated to either supplementation or control groups using a simple randomization procedure in a 1:1 ratio. Four study groups were consequently formed: urban control (UC), urban supplemented (US), rural control (RC), and rural supplemented (RS), with 15 participants in each group. Due to the nature of the intervention, blinding of participants and investigators was not feasible, and placebo capsules were not administered.

Written informed consent was obtained from all participants before enrolment. The study protocol was approved by the Institutional Ethics Committee of Punjab Agricultural University, Ludhiana (Approval No. DR.III.AU.2023/5339–54), and all study procedures were conducted in accordance with the principles of the Declaration of Helsinki.

### 2.2 Sample size consideration

The sample size was determined based on feasibility considerations, participant availability, study duration, resource constraints, and the intensive nature of biochemical, anthropometric, and neurodevelopmental assessments conducted during the intervention period. As this study formed part of a doctoral research project, a total of 60 eligible lactating mothers (15 participants per group) were enrolled and followed longitudinally from 1 to 6 months postpartum. Although a formal *a priori* sample size calculation was not performed, the study was designed as an exploratory intervention trial to generate preliminary evidence regarding the effects of maternal DHA supplementation on breast milk DHA concentration and infant developmental outcomes among urban and rural populations in Punjab.

### 2.3 Supplementation protocol

Participants were categorized into four groups: urban control (UC), urban supplemented (US), rural control (RC), and rural supplemented (RS) (*n* = 15/group). Mothers in the supplemented groups received one soft gelatin capsule daily containing 300 mg docosahexaenoic acid (DHA) and 60 mg eicosapentaenoic acid (EPA) for 150 days, whereas control groups continued their habitual diets without supplementation. Baseline assessments were conducted at 1 month postpartum and follow-up assessments at 6 months postpartum. Similar maternal omega-3 supplementation protocols have been reported previously (11).

### 2.4 Compliance monitoring

To promote adherence to the supplementation protocol, DHA capsules were distributed monthly during scheduled visits at Civil Hospital, Ludhiana. Participants were contacted regularly through weekly follow-up interactions to monitor supplement consumption, address concerns, and encourage continued participation. Compliance was assessed through participant self-report during follow-up visits and communications. All enrolled participants completed the intervention period and attended the scheduled follow-up assessments.

### 2.5 Dietary assessment

Dietary intake was assessed using a 3-day 24-hour dietary recall method (12). Nutrient intake was computed using DietCal software and compared with the Recommended Dietary Allowances proposed by the Indian Council of Medical Research (21). Frequency of consumption of omega-3-rich foods, including fish, flaxseeds, walnuts, mustard oil, nuts, and green leafy vegetables, was also recorded using a food frequency questionnaire.

### 2.6 Anthropometric assessment

Maternal anthropometric measurements, including height, weight, waist circumference, and hip circumference, were recorded using standard procedures (13). Body mass index (BMI), waist-to-hip ratio, and waist-to-height ratio were subsequently calculated. BMI classification was performed according to World Health Organization criteria.

Infant anthropometric measurements, including weight, length, and head circumference, were assessed according to WHO child growth standards (14).

### 2.7 Biochemical analysis

Maternal erythrocyte fatty acid composition and breast milk DHA concentration were assessed at baseline (1 month postpartum) and post-intervention (6 months postpartum). Capillary blood samples were collected on dried blood spot cards, while breast milk samples were collected as dried milk spots using standardized collection procedures. Samples were stored and transported according to laboratory recommendations and analyzed at a NABL-accredited laboratory using gas chromatography techniques for fatty acid profiling.

The proportions of DHA and other fatty acids were expressed as percentages of total identified fatty acids. Quality control procedures were performed by the analytical laboratory to ensure reliability and consistency of measurements.

### 2.8 Neurodevelopmental assessment

Infant neurodevelopment was assessed at 6 months postpartum using the Developmental Assessment Scale for Indian Infants (DASII), the Indian adaptation of the Bayley Scales of Infant Development developed by Phatak (20). The assessment was administered by trained personnel according to standardized procedures outlined in the DASII manual. The scale evaluates motor and mental developmental domains through age-appropriate tasks assessing gross and fine motor skills, language development, cognitive abilities, social interaction, and adaptive behavior.

Developmental age was determined for each infant and used to calculate the Motor Development Quotient (DMoQ) and Mental Development Quotient (DMeQ) according to standard DASII scoring procedures. Higher quotient scores indicate more advanced developmental performance relative to chronological age.

### 2.9 Statistical analysis

Data were analyzed using IBM SPSS Statistics software (Version 2.0; IBM Corp., Armonk, NY, USA). Continuous variables are presented as mean ± standard deviation (SD), while categorical variables are expressed as frequencies and percentages. Data distribution was assessed for normality using the Shapiro–Wilk test before statistical analyses.

Baseline differences between urban and rural participants, as well as among study groups, were evaluated using independent-samples t-tests or one-way analysis of variance (ANOVA), as appropriate. Changes in maternal erythrocyte DHA concentration, breast milk DHA concentration, and infant anthropometric measurements between baseline (1 month postpartum) and post-intervention (6 months postpartum) were assessed using paired Student’s t-tests. Between-group comparisons were performed using one-way ANOVA followed by Tukey’s *post hoc* test for multiple comparisons.

Pearson’s correlation analysis was used to examine associations between maternal erythrocyte DHA concentration, breast milk DHA concentration, and infant neurodevelopmental outcomes, including Motor Development Quotient (DMoQ) and Mental Development Quotient (DMeQ) scores. Ninety-five percent confidence intervals (95% CI) were calculated where appropriate to estimate the precision of effect estimates.

All statistical tests were two-tailed, and statistical significance was established at *p* < 0.05.

## 3 Results

### 3.1 Socio-demographic characteristics of the participants

The socio-demographic characteristics of the urban and rural lactating mothers are presented in Table 1. The mean age of the participants was comparable between urban (23.8 ± 3.9 years; 95% CI: 22.34–25.26) and rural mothers (22.9 ± 3.6 years; 95% CI: 21.56–24.24), with no significant difference observed (*p* = 0.36). Educational attainment differed significantly between the groups, with 10.0% of urban mothers being graduates compared to none among rural mothers (*p* = 0.04). A substantially higher proportion of urban mothers were employed (60.0%; 95% CI: 42.5–77.5) compared with rural mothers (16.0%; 95% CI: 2.9–29.1) (*p* < 0.001). No significant differences were observed between urban and rural participants with respect to dietary preferences, including vegetarianism (53.3% vs. 66.7%; *p* = 0.29) and fish/seafood consumption (20.0% vs. 10.0%; *p* = 0.28). Similarly, the proportion of primiparous mothers was comparable between the two groups (56.7% vs. 60.0%; *p* = 0.80).

**Table 1:** Socio-demographic characteristics of urban and rural lactating mothers.

Characteristic	Urban (*n* = 30) Mean ± SD / %	Urban 95% CI	Rural (*n* = 30) Mean ± SD / %	Rural 95% CI	*p* value
Age (years)	23.8 ± 3.9	22.34–25.26	22.9 ± 3.6	21.56–24.24	0.36
Graduates (%)	10.0	0.0–20.7	0.0	0.0–0.0	0.04*
Working mothers (%)	60.0	42.5–77.5	16.0	2.9–29.1	<0.001***
Vegetarian (%)	53.3	35.5–71.1	66.7	49.8–83.6	0.29
Fish/seafood consumers (%)	20.0	5.7–34.3	10.0	0.0–20.7	0.28
Primiparous mothers (%)	56.7	39.0–74.4	60.0	42.5–77.5	0.80

Values are presented as mean ± standard deviation (SD) or percentages (%). Ninety-five percent confidence intervals (95% CI) are reported for continuous and categorical variables. Differences between urban and rural participants were assessed using independent-samples t-tests for continuous variables and Chi-square tests for categorical variables. Statistical significance was established at *p* < 0.05. *p < 0.05; ** *p* < 0.01; *** *p* < 0.001.

### 3.2 Baseline anthropometric characteristics of lactating mothers

Baseline anthropometric characteristics of the study participants are presented in Table 2. Significant differences were observed between urban and rural mothers for all anthropometric parameters (*p* < 0.001). Rural mothers exhibited higher body weight, BMI, waist circumference, and waist-to-height ratio (WHtR) compared with urban mothers. Mean body weight ranged from 50.8 ± 2.9 kg (95% CI: 49.19–52.41) in the urban supplemented group to 61.4 ± 2.5 kg (95% CI: 60.02–62.78) in the rural supplemented group. Similarly, BMI values were higher among rural participants (24.5–24.8 kg/m^2^) compared with urban participants (20.8–21.0 kg/m^2^), with narrow confidence intervals indicating consistency within groups. Waist circumference was also significantly greater among rural mothers, ranging from 95.8 ± 0.4 cm (95% CI: 95.58–96.02) in the rural control group to 96.5 ± 0.3 cm (95% CI: 96.33–96.67) in the rural supplemented group, compared with 87.6–88.0 cm among urban participants. Likewise, WHtR values were higher among rural mothers (0.61–0.62) than urban mothers (0.56–0.57), reflecting greater central adiposity among the rural study population. No meaningful differences were observed between control and supplemented groups within the same geographical setting at baseline, indicating comparability prior to intervention.

**Table 2:** Baseline anthropometric characteristics of lactating mothers.

Parameter	UC (*n* = 15) Mean ± SD	UC 95% CI	US (*n* = 15) Mean ± SD	US 95% CI	RC (*n* = 15) Mean ± SD	RC 95% CI	RS (*n* = 15) Mean ± SD	RS 95% CI	*p*-value
Weight (kg)	51.2 ± 2.6	49.76–52.64	50.8 ± 2.9	49.19–52.41	60.9 ± 2.8	59.35–62.45	61.4 ± 2.5	60.02–62.78	<0.001***
BMI (kg/m^2^)	21.0 ± 1.1	20.39–21.61	20.8 ± 1.2	20.14–21.46	24.5 ± 1.0	23.95–25.05	24.8 ± 1.1	24.19–25.41	<0.001***
Waist circumference (cm)	87.6 ± 0.5	87.32–87.88	88.0 ± 0.4	87.78–88.22	95.8 ± 0.4	95.58–96.02	96.5 ± 0.3	96.33–96.67	<0.001***
WHtR	0.56 ± 0.02	0.549–0.571	0.57 ± 0.01	0.564–0.576	0.61 ± 0.01	0.604–0.616	0.62 ± 0.01	0.614–0.626	<0.001***

Values are presented as mean ± standard deviation (SD). Ninety-five percent confidence intervals (95% CI) were calculated around the mean values. Differences among study groups were assessed using one-way analysis of variance (ANOVA) followed by Tukey’s *post hoc* test. Statistical significance was established at *p* < 0.05. ***p < 0.001. UC, Urban Control; US, Urban Supplemented; RC, Rural Control; RS, Rural Supplemented; BMI, Body Mass Index; WHtR, Waist-to-Height Ratio; CI, Confidence Interval.

### 3.3 Dietary intake and adequacy of omega-3 fatty acids

The dietary intake of omega-3 fatty acids among urban and rural lactating mothers is presented in Table 3. Urban mothers reported significantly higher intakes of alpha-linolenic acid (ALA), docosahexaenoic acid (DHA), and eicosapentaenoic acid (EPA) compared with their rural counterparts (*p* < 0.001). Mean ALA intake was 780 ± 65 mg/day (95% CI: 755.8–804.2) among urban mothers and 640 ± 58 mg/day (95% CI: 618.4–661.6) among rural mothers, corresponding to 55.7 and 45.7% of the recommended intake, respectively. Similarly, DHA intake was significantly higher in urban mothers (42 ± 10 mg/day; 95% CI: 38.3–45.7) than in rural mothers (28 ± 8 mg/day; 95% CI: 25.0–31.0), although both groups consumed substantially lower amounts than the recommended intake of 200 mg/day, achieving only 21.0 and 14.0% adequacy, respectively.

**Table 3:** Dietary intake and adequacy of omega-3 fatty acids among urban and rural lactating mothers.

Nutrient	Urban (*n* = 30) Mean ± SD	Urban 95% CI	Rural (*n* = 30) Mean ± SD	Rural 95% CI	RDA/AI* (mg/day)	Adequacy (%)	p-value
ALA (mg/day)	780 ± 65	755.8–804.2	640 ± 58	618.4–661.6	1,400	55.7 vs. 45.7	<0.001***
DHA (mg/day)	42 ± 10	38.3–45.7	28 ± 8	25.0–31.0	200	21.0 vs. 14.0	<0.001***
EPA (mg/day)	15 ± 5	13.1–16.9	9 ± 4	7.5–10.5	—	—	<0.001***

Values are expressed as mean ± standard deviation (SD). Ninety-five percent confidence intervals (95% CI) were calculated around the mean values. Adequacy (%) was calculated as the percentage contribution of mean intake relative to the recommended dietary allowance (RDA) or adequate intake (AI). Differences between urban and rural participants were assessed using independent-samples t-tests. Statistical significance was established at *p* < 0.05. ***p < 0.001. * RDA/AI values were based on current dietary recommendations for lactating women.

EPA intake also differed significantly between groups, with urban mothers consuming 15 ± 5 mg/day (95% CI: 13.1–16.9) compared with 9 ± 4 mg/day (95% CI: 7.5–10.5) among rural mothers (*p* < 0.001). Overall, the findings indicate inadequate dietary intake of long-chain omega-3 fatty acids among lactating mothers, with comparatively lower intake and adequacy observed in rural participants.

### 3.4 Maternal erythrocyte DHA concentration

Changes in maternal erythrocyte DHA concentration following supplementation are presented in Table 4. At baseline, erythrocyte DHA concentrations were comparable across study groups, ranging from 1.9 ± 0.2% (95% CI: 1.79–2.01) in the rural control group to 2.2 ± 0.2% (95% CI: 2.09–2.31) in the urban supplemented group. Following 150 days of supplementation, substantial increases in erythrocyte DHA concentration were observed among mothers receiving DHA supplementation, whereas only minimal changes were noted in the control groups.

**Table 4:** Maternal erythrocyte DHA concentration before and after supplementation.

Group	Baseline (% of total fatty acids) Mean ± SD	Baseline 95% CI	6 Months (% of total fatty acids) Mean ± SD	6 Months 95% CI	Mean difference	% Change	*p*-value
Urban control	2.1 ± 0.3	1.93–2.27	2.2 ± 0.2	2.09–2.31	+0.1	+4.8	0.312
Urban supplemented	2.2 ± 0.2	2.09–2.31	4.8 ± 0.4	4.58–5.02	+2.6	+118.2	<0.001***
Rural control	1.9 ± 0.2	1.79–2.01	2.0 ± 0.3	1.83–2.17	+0.1	+5.3	0.285
Rural supplemented	2.0 ± 0.3	1.83–2.17	4.2 ± 0.3	4.03–4.37	+2.2	+110.0	<0.001***

Values are expressed as mean ± standard deviation (SD) (n = 15 mothers per group). Erythrocyte DHA concentration is expressed as percentage of total fatty acids. Baseline measurements were obtained at 1 month postpartum and post-intervention measurements at 6 months postpartum. Ninety-five percent confidence intervals (95% CI) were calculated around the mean values. Within-group comparisons were performed using paired Student’s t-tests. Statistical significance was established at *p* < 0.05. ***p < 0.001. DHA, Docosahexaenoic Acid; CI, Confidence Interval.

The urban supplemented group demonstrated the greatest increase, with erythrocyte DHA concentration rising from 2.2 ± 0.2% to 4.8 ± 0.4% (95% CI: 4.58–5.02), representing an increase of 118.2% (*p* < 0.001). Similarly, the rural supplemented group exhibited a significant increase from 2.0 ± 0.3% to 4.2 ± 0.3% (95% CI: 4.03–4.37), corresponding to a 110.0% increase (*p* < 0.001). In contrast, only marginal, non-significant increases were observed in the urban control (+4.8%; *p* = 0.312) and rural control groups (+5.3%; *p* = 0.285). These findings indicate that maternal DHA supplementation markedly improved erythrocyte DHA status in both urban and rural lactating mothers.

### 3.5 Breast milk DHA concentration

Changes in breast milk DHA concentration following maternal supplementation are presented in Table 5. Baseline breast milk DHA concentrations were low and comparable across all study groups, ranging from 0.19 ± 0.02% (95% CI: 0.18–0.20) in the rural supplemented group to 0.24 ± 0.04% (95% CI: 0.22–0.26) in the urban control group. Following 150 days of supplementation, marked increases in breast milk DHA concentration were observed among supplemented mothers, whereas only minimal changes were evident in the control groups.

**Table 5:** Breast milk DHA concentration before and after supplementation.

Group	Baseline (% of total fatty acids) Mean ± SD	Baseline 95% CI	6 Months (% of total fatty acids) Mean ± SD	6 Months 95% CI	Mean Difference	% Change	p-value
Urban control	0.24 ± 0.04	0.22–0.26	0.25 ± 0.03	0.23–0.27	+0.01	+4.2	0.411
Urban supplemented	0.23 ± 0.03	0.21–0.25	0.68 ± 0.05	0.65–0.71	+0.45	+195.7	<0.001***
Rural control	0.20 ± 0.03	0.18–0.22	0.21 ± 0.03	0.19–0.23	+0.01	+5.0	0.382
Rural supplemented	0.19 ± 0.02	0.18–0.20	0.59 ± 0.04	0.57–0.61	+0.40	+210.5	<0.001***

Values are expressed as mean ± standard deviation (SD) (*n* = 15 mothers per group). Breast milk DHA concentration is expressed as percentage of total fatty acids. Milk samples were collected at baseline (1 month postpartum) and post-intervention (6 months postpartum). Ninety-five percent confidence intervals (95% CI) were calculated around the mean values. Within-group comparisons were performed using paired Student’s *t*-tests. Statistical significance was established at *p* < 0.05. ***p < 0.001. DHA, Docosahexaenoic Acid; CI, Confidence Interval.

The urban supplemented group demonstrated a significant increase in breast milk DHA concentration from 0.23 ± 0.03% to 0.68 ± 0.05% (95% CI: 0.65–0.71), representing an increase of 195.7% (*p* < 0.001). Similarly, breast milk DHA concentration in the rural supplemented group increased from 0.19 ± 0.02% to 0.59 ± 0.04% (95% CI: 0.57–0.61), corresponding to a 210.5% increase (*p* < 0.001). In contrast, only small, non-significant increases were observed in the urban control (+4.2%; *p* = 0.411) and rural control groups (+5.0%; *p* = 0.382). These findings indicate that maternal DHA supplementation was associated with substantially higher breast milk DHA concentrations in both urban and rural lactating mothers.

### 3.6 Infant anthropometric outcomes

Infant anthropometric outcomes at 6 months postpartum are presented in Table 6. Infants born to supplemented mothers exhibited significantly greater weight, length, and head circumference compared with infants in the respective control groups. In the urban population, mean body weight was significantly higher among infants of supplemented mothers (7.4 ± 0.4 kg; 95% CI: 7.18–7.62) than among infants in the urban control group (6.8 ± 0.5 kg; 95% CI: 6.52–7.08) (*p* = 0.018). Similarly, infants in the rural supplemented group had significantly greater body weight (7.1 ± 0.5 kg; 95% CI: 6.82–7.38) compared with those in the rural control group (6.5 ± 0.4 kg; 95% CI: 6.28–6.72) (*p* = 0.026).

**Table 6:** Infant anthropometric outcomes at sixth month postpartum.

Parameter	Group (*n* = 15)	Mean ± SD	95% CI	p-value†
Weight (kg)	Urban control	6.8 ± 0.5	6.52–7.08	—
	Urban supplemented	7.4 ± 0.4	7.18–7.62	0.018*
	Rural control	6.5 ± 0.4	6.28–6.72	—
	Rural supplemented	7.1 ± 0.5	6.82–7.38	0.026*
Length (cm)	Urban control	64.2 ± 1.8	63.20–65.20	—
	Urban supplemented	66.8 ± 1.7	65.86–67.74	0.014*
	Rural control	63.1 ± 1.6	62.21–63.99	—
	Rural supplemented	65.5 ± 1.5	64.67–66.33	0.022*
Head circumference (cm)	Urban control	41.8 ± 0.9	41.30–42.30	—
	Urban supplemented	43.2 ± 0.8	42.76–43.64	0.011*
	Rural control	41.0 ± 0.8	40.56–41.44	—
	Rural supplemented	42.6 ± 0.7	42.21–42.99	0.019*

Values are expressed as mean ± standard deviation (SD) (*n* = 15 infants per group). Anthropometric measurements were obtained at 6 months postpartum according to World Health Organization (WHO) child growth assessment procedures. The 95% confidence intervals (CI) were calculated around the mean values. Group comparisons were performed using one-way ANOVA followed by Tukey’s *post hoc* test. † Comparison between supplemented and respective control groups within the same population. *p < 0.05. CI, Confidence Interval; SD, Standard Deviation; UC, Urban Control; US, Urban Supplemented; RC, Rural Control; RS, Rural Supplemented.

A comparable trend was observed for recumbent length. Infants in the urban supplemented group attained a mean length of 66.8 ± 1.7 cm (95% CI: 65.86–67.74), which was significantly greater than that of the urban control group (64.2 ± 1.8 cm; 95% CI: 63.20–65.20) (*p* = 0.014). Likewise, infants in the rural supplemented group demonstrated greater length (65.5 ± 1.5 cm; 95% CI: 64.67–66.33) than those in the rural control group (63.1 ± 1.6 cm; 95% CI: 62.21–63.99) (*p* = 0.022).

Head circumference measurements also differed significantly between supplemented and control groups. Infants of supplemented mothers exhibited larger head circumference values in both urban (43.2 ± 0.8 cm; 95% CI: 42.76–43.64 vs. 41.8 ± 0.9 cm; 95% CI: 41.30–42.30; *p* = 0.011) and rural settings (42.6 ± 0.7 cm; 95% CI: 42.21–42.99 vs. 41.0 ± 0.8 cm; 95% CI: 40.56–41.44; *p* = 0.019). Overall, infants whose mothers received DHA supplementation demonstrated significantly higher anthropometric measurements at 6 months postpartum compared with infants in the corresponding control groups.

### 3.7 WHO growth indicators and conditional weight gain

WHO growth indicators and conditional weight gain classifications of infants at 6 months postpartum are presented in Table 7. Infants in the supplemented groups demonstrated more favorable growth indicator scores compared with their respective control groups. Mean weight-for-age Z-scores (WAZ) were higher among supplemented infants, with values of 0.18 ± 0.61 in the urban supplemented group and −0.05 ± 0.65 in the rural supplemented group, compared with −0.42 ± 0.68 and −0.71 ± 0.74 in the corresponding urban and rural control groups, respectively (*p* = 0.032).

**Table 7:** WHO growth indicators of infants at sixth month postpartum.

Indicator	Urban control (*n* = 15)	Urban supplemented (*n* = 15)	Rural control (*n* = 15)	Rural supplemented (*n* = 15)	*p*-value
Weight-for-age Z-score (WAZ)	−0.42 ± 0.68	0.18 ± 0.61	−0.71 ± 0.74	−0.05 ± 0.65	0.032*
Length-for-age Z-score (LAZ)	−0.58 ± 0.72	0.02 ± 0.67	−0.89 ± 0.81	−0.22 ± 0.69	0.028*
Weight-for-length Z-score (WLZ)	−0.18 ± 0.64	0.41 ± 0.58	−0.36 ± 0.69	0.20 ± 0.60	0.041*
Conditional weight gain score	−0.12 ± 0.58	0.48 ± 0.62	−0.25 ± 0.63	0.35 ± 0.57	0.036*

Classification of conditional weight gain				
Category	Urban control *n* (%)	Urban supplemented *n* (%)	Rural control *n* (%)	Rural supplemented *n* (%)
Slow weight gain (< −0.67 SD)	3 (20.0)	1 (6.7)	4 (26.7)	2 (13.3)
Normal weight gain (−0.67 to 0.67 SD)	10 (66.7)	12 (80.0)	9 (60.0)	11 (73.3)
Rapid weight gain (> 0.67 SD)	2 (13.3)	2 (13.3)	2 (13.3)	2 (13.3)

Values are expressed as mean ± standard deviation (SD) (*n* = 15 infants per group). Weight-for-age Z-score (WAZ), length-for-age Z-score (LAZ), and weight-for-length Z-score (WLZ) were evaluated using World Health Organization (WHO) Child Growth Standards. Conditional weight gain was classified as slow weight gain (SWG; < −0.67 SD), normal weight gain (NWG; −0.67 to 0.67 SD), and rapid weight gain (RWG; > 0.67 SD). Group comparisons were performed using one-way analysis of variance (ANOVA) followed by Tukey’s post hoc test. Statistical significance was established at p < 0.05. *p < 0.05. WAZ, Weight-for-Age Z-score; LAZ, Length-for-Age Z-score; WLZ, Weight-for-Length Z-score; SD, Standard Deviation; UC, Urban Control; US, Urban Supplemented; RC, Rural Control; RS, Rural Supplemented.

Similarly, length-for-age Z-scores (LAZ) were higher among supplemented infants than controls. The urban supplemented group exhibited a mean LAZ of 0.02 ± 0.67, while the rural supplemented group had a mean LAZ of −0.22 ± 0.69, compared with −0.58 ± 0.72 and −0.89 ± 0.81 among urban and rural controls, respectively (*p* = 0.028). Weight-for-length Z-scores (WLZ) also differed significantly across study groups (*p* = 0.041), with positive mean values observed among supplemented infants, indicating comparatively better weight status relative to length.

Conditional weight gain scores were significantly higher among supplemented groups than controls (*p* = 0.036). A greater proportion of infants in the supplemented groups were classified within the normal weight gain category (80.0% in urban supplemented and 73.3% in rural supplemented groups) compared with control groups (66.7% in urban control and 60.0% in rural control groups). Conversely, slow weight gain was more frequently observed among control infants, particularly in the rural control group (26.7%). The proportion of infants classified as rapid weight gain was similar across study groups.

Overall, the growth indicator analysis suggested more favorable growth patterns among infants whose mothers received DHA supplementation during lactation compared with infants in the corresponding control groups.

### 3.8 Infant neurodevelopmental outcomes

Infant neurodevelopmental outcomes assessed using the Developmental Assessment Scale for Indian Infants (DASII) at 6 months postpartum are presented in Table 8. Infants whose mothers received DHA supplementation achieved higher Motor Development Quotient (DMoQ) and Mental Development Quotient (DMeQ) scores compared with infants in the corresponding control groups.

**Table 8:** Neurodevelopmental outcomes of infants at sixth month postpartum.

Domain	Group (*n* = 15)	Mean ± SD	95% CI	Severe delay (≤70) *n* (%)	Mild delay (71–84) *n* (%)	Normal (≥85) *n* (%)	Within-group *p*-value†	vs. urban control‡	vs. rural control‡	vs. respective control§
Motor development quotient (DMoQ)	Urban control	89.6 ± 4.2	87.27–91.93	0 (0.0)	3 (20.0)	12 (80.0)	—	—	0.048*	—
	Urban supplemented	102.8 ± 5.1	99.98–105.62	0 (0.0)	1 (6.7)	14 (93.3)	<0.001	<0.001	<0.001	<0.001
	Rural control	86.2 ± 4.0	83.98–88.42	0 (0.0)	4 (26.7)	11 (73.3)	—	0.048*	—	—
	Rural supplemented	98.5 ± 4.8	95.84–101.16	0 (0.0)	1 (6.7)	14 (93.3)	<0.001	<0.001	<0.001	<0.001
Mental development quotient (DMeQ)	Urban control	91.1 ± 4.4	88.66–93.54	0 (0.0)	2 (13.3)	13 (86.7)	—	—	0.046*	—
	Urban supplemented	105.3 ± 5.4	102.31–108.29	0 (0.0)	1 (6.7)	14 (93.3)	<0.001	<0.001	<0.001	<0.001
	Rural control	87.5 ± 4.2	85.17–89.83	0 (0.0)	3 (20.0)	12 (80.0)	—	0.046*	—	—
	Rural supplemented	100.4 ± 5.0	97.63–103.17	0 (0.0)	1 (6.7)	14 (93.3)	<0.001	<0.001	<0.001	<0.001

Values are expressed as mean ± standard deviation (SD) (n = 15 infants per group). Ninety-five percent confidence intervals (95% CI) were calculated around the mean values. Neurodevelopment was assessed at 6 months postpartum using the Developmental Assessment Scale for Indian Infants (DASII). Development Quotient (DQ) scores were categorized as severe delay (≤70), mild delay (71–84), and normal development (≥85). Group comparisons were performed using one-way ANOVA followed by Tukey’s post hoc test. † Within-group comparison against the normative developmental score. ‡ Comparison with Urban Control group. § Comparison with the respective control group within the same geographical setting. *p < 0.05; **p < 0.01; ***p < 0.001. DASII, developmental assessment scale for Indian infants; DMoQ, motor development quotient; DMeQ, mental development quotient; CI, confidence interval.

For motor development, mean DMoQ scores ranged from 86.2 ± 4.0 (95% CI: 83.98–88.42) in the rural control group to 102.8 ± 5.1 (95% CI: 99.98–105.62) in the urban supplemented group. Both supplemented groups demonstrated significantly higher motor development scores than their respective controls (*p* < 0.001). Similarly, mental development scores were higher among infants in the supplemented groups, with mean DMeQ scores of 105.3 ± 5.4 (95% CI: 102.31–108.29) in the urban supplemented group and 100.4 ± 5.0 (95% CI: 97.63–103.17) in the rural supplemented group, compared with 91.1 ± 4.4 (95% CI: 88.66–93.54) and 87.5 ± 4.2 (95% CI: 85.17–89.83) in the corresponding urban and rural control groups, respectively (*p* < 0.001).

Categorical assessment of developmental status revealed that more than 93% of infants in both supplemented groups were classified within the normal developmental range (≥85) for both motor and mental development. In contrast, a higher proportion of infants in the control groups were classified within the mild delay category (71–84), particularly among rural controls. No infant in any study group was classified as having severe developmental delay (≤70).

Overall, maternal DHA supplementation was associated with higher developmental quotient scores and a greater proportion of infants achieving normal developmental status at 6 months postpartum.

### 3.9 Association between breast milk DHA and infant neurodevelopment

**Figure 2:**
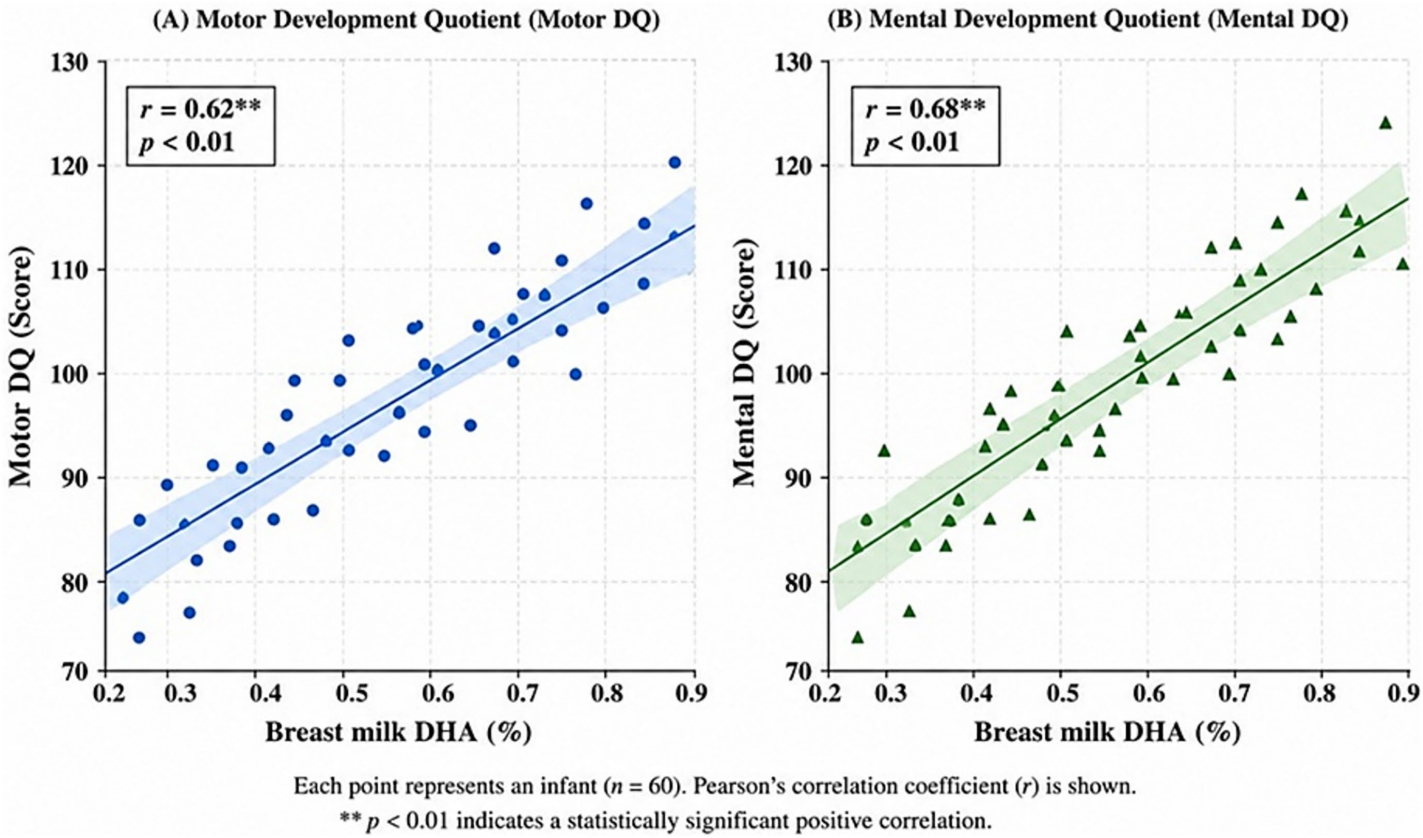
Correlation between breast milk DHA concentration and infant neurodevelopmental outcomes. **(A)** Association between breast milk DHA (%) and Motor Development Quotient (DMoQ). **(B)** Association between breast milk DHA (%) and Mental Development Quotient (DMeQ). Each point represents an individual infant (*n* = 60). Pearson’s correlation coefficients (r) and corresponding significance levels are shown.

Breast milk DHA concentration demonstrated significant positive associations with infant neurodevelopmental outcomes. Breast milk DHA was positively correlated with both DMoQ (*r* = 0.62, *p* < 0.01) and DMeQ (*r* = 0.67, *p* < 0.01), suggesting that improved maternal DHA status contributed to enhanced infant motor and cognitive development (Figure 2).

## 4 Discussion

The present study investigated the effects of maternal DHA supplementation during lactation on maternal DHA status, breast milk DHA concentration, infant growth, and neurodevelopment among urban and rural populations in Punjab, India. The findings demonstrated inadequate dietary intake of omega-3 fatty acids among lactating mothers, particularly among rural participants, and showed that maternal DHA supplementation was associated with significant improvements in maternal erythrocyte DHA concentration, breast milk DHA content, infant growth indicators, and neurodevelopmental outcomes.

Dietary assessment revealed inadequate intake of omega-3 fatty acids among both urban and rural mothers, with significantly lower intakes observed among rural participants. The diets of most mothers were predominantly cereal-based and characterized by limited consumption of fish, flaxseeds, walnuts, and other omega-3-rich foods. Similar findings have been reported in Indian populations, where low seafood consumption and greater reliance on omega-6-rich vegetable oils contribute to poor DHA status among women of reproductive age (15, 16). Rural mothers also demonstrated lower dietary diversity, which may partly explain the reduced adequacy of alpha-linolenic acid (ALA) and DHA intake observed in the present study. These findings highlight the continuing challenge of achieving adequate omega-3 fatty acid intake among lactating women in resource-constrained settings.

Maternal anthropometric assessment revealed significantly higher BMI, waist circumference, and waist-to-height ratio among rural mothers compared with urban participants. These findings are consistent with the growing evidence of the double burden of malnutrition in India, where overweight and obesity coexist with micronutrient inadequacies (17). Rural mothers exhibited greater central adiposity despite lower dietary diversity and poorer omega-3 intake, suggesting that dietary quality rather than caloric adequacy may be a critical determinant of nutritional status in these populations. Differences in postpartum dietary practices, physical activity levels, socioeconomic status, and healthcare access may have contributed to the observed rural–urban disparities.

Maternal DHA supplementation resulted in substantial improvements in erythrocyte DHA concentration, indicating effective incorporation of DHA into maternal tissues and enhanced long-term omega-3 status. Erythrocyte DHA concentrations more than doubled among supplemented mothers, whereas only minimal, non-significant changes were observed in control groups. These findings are consistent with previous studies demonstrating significant increases in maternal erythrocyte DHA following omega-3 supplementation during pregnancy and lactation (5, 18). Since erythrocyte DHA is considered a reliable biomarker of long-term DHA status, the observed increases provide evidence of the biological effectiveness of supplementation in this population.

Breast milk DHA concentration increased significantly following supplementation, confirming the direct influence of maternal dietary intake on breast milk fatty acid composition. Because endogenous conversion of alpha-linolenic acid to DHA is limited in humans, maternal diet remains the primary determinant of breast milk DHA concentration (4). The substantial increases in breast milk DHA observed among supplemented mothers are consistent with previous intervention studies reporting enhanced milk DHA concentrations following maternal fish oil or DHA supplementation (3, 19). Lower baseline breast milk DHA concentrations among rural mothers may reflect poorer dietary omega-3 intake, lower dietary diversity, and reduced access to DHA-rich foods. Collectively, these findings emphasize the responsiveness of breast milk fatty acid composition to maternal nutritional interventions during lactation.

In addition to improving maternal DHA status, supplementation was associated with more favorable infant growth outcomes. Infants of supplemented mothers demonstrated higher weight, length, and head circumference measurements at 6 months postpartum compared with infants in the corresponding control groups. Furthermore, growth trajectory analysis using WHO growth indicators revealed higher weight-for-age (WAZ), length-for-age (LAZ), and weight-for-length (WLZ) Z-scores among supplemented infants. Conditional weight gain analysis also demonstrated a greater proportion of infants within the normal growth category and a lower prevalence of slow weight gain among supplemented groups. These findings suggest that improved maternal DHA status and enhanced breast milk DHA concentration may contribute to more favorable infant growth patterns during early life.

DHA plays important roles in membrane structure, cellular signaling, inflammatory regulation, and metabolic functioning, all of which may influence infant growth and development (8). The observed improvements in growth indicators are biologically plausible and are consistent with studies reporting associations between maternal omega-3 status and infant growth outcomes (5, 18). Nevertheless, infant growth is influenced by multiple determinants, including genetics, feeding practices, maternal health, environmental conditions, and socioeconomic factors. Consequently, the growth-related findings should be interpreted cautiously, particularly given the relatively small sample size and limited follow-up period.

The present study also observed higher neurodevelopmental scores among infants whose mothers received DHA supplementation during lactation. Infants in the supplemented groups achieved significantly higher Motor Development Quotient (DMoQ) and Mental Development Quotient (DMeQ) scores compared with infants in the control groups. In addition, a greater proportion of supplemented infants were classified within the normal developmental range, while fewer infants exhibited mild developmental delay. DHA is highly concentrated within neuronal membranes and retinal tissues and plays a critical role in synaptogenesis, neurotransmission, myelination, and visual processing during infancy (1, 2). Increased DHA availability through breast milk may therefore support neurodevelopment during this period of rapid brain growth and maturation.

The neurodevelopmental findings observed in the present study are consistent with previous investigations reporting beneficial associations between maternal omega-3 supplementation and infant cognitive and motor development (5–7, 18). A systematic review by Shulkin et al. (10) similarly concluded that maternal omega-3 supplementation may positively influence attention, memory, and early cognitive performance. Although urban supplemented infants demonstrated slightly higher developmental scores than rural supplemented infants, these differences may reflect broader contextual influences, including maternal education, household environment, dietary quality, healthcare access, and opportunities for developmental stimulation, rather than supplementation alone.

Breast milk DHA concentration was positively correlated with infant motor and mental developmental quotients, indicating that higher maternal and breast milk DHA status was associated with improved neurodevelopmental performance. These findings support previous evidence linking breast milk DHA with infant cognitive function, neuronal maturation, and visual development (1, 2). Since the first 2 years of life represent a critical window for brain development, ensuring adequate DHA availability during infancy may have important implications for long-term cognitive and behavioral outcomes. However, the observed associations should not be interpreted as evidence of causality, as neurodevelopment is influenced by numerous biological, environmental, and socioeconomic factors.

The findings of the present study have important public health implications for populations with low habitual DHA intake. Maternal omega-3 consumption among Indian lactating women remains substantially below recommended levels because of poor dietary diversity, limited fish consumption, and inadequate nutritional awareness. The results suggest that maternal DHA supplementation during lactation may represent a practical nutritional strategy for improving maternal DHA status and supporting optimal infant growth and neurodevelopment in populations with low omega-3 intake. Promotion of locally available omega-3-rich foods, including flaxseeds, walnuts, mustard oil, and garden cress seeds, together with targeted nutrition education during pregnancy and lactation, may further contribute to improving maternal and infant nutritional outcomes.

### 4.1 Strengths and limitations

The present study has several strengths. First, it evaluated multiple dimensions of maternal and infant health simultaneously, including maternal dietary intake, erythrocyte DHA status, breast milk DHA concentration, infant anthropometric outcomes, WHO growth indicators, and neurodevelopmental performance. Such a comprehensive assessment provides a broader understanding of the potential influence of maternal DHA supplementation during lactation. Second, the inclusion of both urban and rural populations enabled the examination of socioeconomic and dietary differences that may influence maternal DHA status and infant outcomes. Third, infant neurodevelopment was assessed using the Developmental Assessment Scale for Indian Infants (DASII), a validated tool widely used in the Indian context. Finally, the study incorporated objective biochemical markers of DHA status, including erythrocyte and breast milk DHA concentrations, thereby strengthening the biological relevance of the findings.

Despite these strengths, several limitations should be acknowledged. The study included a relatively small sample size, which was determined based on feasibility and participant availability during the conduct of the doctoral research. Consequently, the statistical power to detect smaller effects may have been limited, and the findings should be interpreted with caution. Although participants were randomly allocated to study groups, placebo supplementation and participant blinding were not feasible because of the nature of the intervention, which may have introduced the possibility of performance bias. Furthermore, neurodevelopment is influenced by numerous factors, including maternal education, household environment, socioeconomic status, infant stimulation, healthcare access, and feeding practices, which may not have been fully controlled for in the present study.

The follow-up period was limited to 6 months postpartum; therefore, the long-term effects of maternal DHA supplementation on child growth, cognition, and behavioral outcomes could not be assessed. Although WHO growth indicators and conditional weight gain analyses were incorporated to provide a more comprehensive evaluation of infant growth trajectories, larger longitudinal studies with repeated assessments are required to confirm these findings. Finally, the study was conducted in selected urban and rural communities of Punjab, India, and therefore the findings may not be fully generalizable to other populations with different dietary patterns, socioeconomic conditions, or healthcare systems.

Overall, the results provide preliminary evidence that maternal DHA supplementation during lactation may improve maternal DHA status, increase breast milk DHA concentration, and support favorable infant growth and neurodevelopmental outcomes in populations with low habitual omega-3 fatty acid intake.

## 5 Conclusion

The present study demonstrated inadequate dietary intake of omega-3 fatty acids among lactating mothers in both urban and rural populations, with comparatively lower intake observed among rural participants. Maternal DHA supplementation during lactation was associated with significant increases in erythrocyte DHA concentration and breast milk DHA content, indicating improved maternal DHA status. Infants of supplemented mothers exhibited more favorable anthropometric measurements, improved WHO growth indicators, and higher Motor Development Quotient and Mental Development Quotient scores compared with infants in the corresponding control groups. Positive associations between breast milk DHA concentration and infant neurodevelopmental outcomes further support the importance of adequate maternal DHA status during early infancy.

These findings suggest that maternal DHA supplementation during lactation may represent a useful nutritional strategy for improving maternal DHA status and supporting optimal infant growth and neurodevelopment in populations with low habitual omega-3 fatty acid intake. However, given the relatively small sample size and short follow-up duration, larger longitudinal studies are warranted to confirm these findings and evaluate the long-term effects of maternal DHA supplementation on child growth, cognitive development, and health outcomes.

## Data Availability

The original contributions presented in the study are included in the article/supplementary material, further inquiries can be directed to the corresponding author.
